# Cost and accuracy of advanced breeding trial designs in apple

**DOI:** 10.1038/hortres.2016.8

**Published:** 2016-03-23

**Authors:** Julia M Harshman, Kate M Evans, Craig M Hardner

**Affiliations:** 1 Department of Horticulture, Washington State University Tree Fruit Research and Extension Center, Wenatchee, WA 98801, USA; 2 Queensland Alliance for Agriculture and Food Innovation, University of Queensland, St Lucia, Queensland 4072, Australia

## Abstract

Trialing advanced candidates in tree fruit crops is expensive due to the long-term nature of the planting and labor-intensive evaluations required to make selection decisions. How closely the trait evaluations approximate the true trait value needs balancing with the cost of the program. Designs of field trials of advanced apple candidates in which reduced number of locations, the number of years and the number of harvests per year were modeled to investigate the effect on the cost and accuracy in an operational breeding program. The aim was to find designs that would allow evaluation of the most additional candidates while sacrificing the least accuracy. Critical percentage difference, response to selection, and correlated response were used to examine changes in accuracy of trait evaluations. For the quality traits evaluated, accuracy and response to selection were not substantially reduced for most trial designs. Risk management influences the decision to change trial design, and some designs had greater risk associated with them. Balancing cost and accuracy with risk yields valuable insight into advanced breeding trial design. The methods outlined in this analysis would be well suited to other horticultural crop breeding programs.

## Introduction

New apple (*Malus*×*domestica* Borkh.) cultivars sustain consumer interest and increase industry profitability through improved returns or reduced costs relative to current varieties.^1^ Success of varieties released in recent decades (that is, ‘Honeycrisp’, ‘Fuji’, ‘Cripps Pink’) is largely due to their superior eating quality;^1,2^ improved quality is the aim of many apple breeding programs in order to produce successful new varieties.

Apple improvement programs typically operate a multi-stage selection scheme.^3–5^ The first stage involves evaluation of a large number of un-replicated seedlings. In subsequent stages, the reduced number of selected candidates are clonally propagated and planted in replicated trials, which enable comparisons of genetic potential between candidate selections and current varieties. Multiple traits underlie fruit quality and thus, the decision to advance a candidate. Clonal propagation allows both the additive and non-additive genetic variation to be targeted by selection. Identifying candidates superior to current cultivars is a function of the size of the selection population and the accuracy with which the available data predicts the genetic potential of a candidate selection.^6^ Design of the field trial impacts the accuracy with which traits are evaluated and the breeding program cost.

Accurate prediction of genetic potential in selection environments highly correlated with future commercial planting environments underpins the successful adoption of new varieties as it improves confidence in the genetic potential of the candidate selection.^7^ In apple, these predictions are achieved by trialing clonal replicates over multiple years in multiple locations, usually with multiple blocks within location; accuracy is improved with increased replication. However, maintaining replicated trials of clonal apple candidates is expensive,^8^ and there are trade-offs between maximizing accuracy and minimizing cost to the program with the limited resources available. Several evaluation criteria can give insight into which trial design factors most influence accuracy and those changes in accuracy can then be compared against changes in the breeding program cost.

Critical percentage difference (CPD) is a measure of accuracy that estimates the observed percentage difference needed to claim that a selection and a control are different with a confidence of *α*, similar to the least significant difference statistic.^9–14^ CPD is a function of the percentage s.e. of the estimate of the true mean difference recorded in the trial and the value from the standardized normal distribution that is exceeded with a probability of *α*.

Response to selection (RS) is the predicted gain from directional selection.^6^ It can be used to evaluate trials of differing sizes^9^ as variation in numbers of entries may affect both the selection intensity and accuracy of the predicted candidate effect. With clonal replicates, it can be used to explore how changes in trial design factors alter the average trait value of a population from one stage to the next. RS is a function of selection intensity, accuracy of prediction of genetic potential in selection environments, and the correlation between genetic potential in selection environments and future commercial planting environments.^15^

Correlated response to selection (CRS) indicates the magnitude of directional gain that can be achieved in one trait when selection is applied to a second, correlated trait. Indirect selection can be more effective than direct selection when the second trait is highly correlated and can be evaluated more quickly, with less cost or with greater accuracy.^6^ Using instrumental traits to improve sensory traits is of interest as they are typically less expensive to measure, eliminate problems with ‘taster-fatigue’^16,17^ and the heritability of instrumental measures may be higher than that for the sensory trait.^17,18^ Gain from indirect selection is strongly dependent on the correlation between traits. In this study, CRS is used to evaluate the directional progress that can be made in a sensory fruit quality trait when selection is applied to the correlated instrumental fruit quality trait. As with RS, CRS can be used to explore how changes in trial design factors alter the average trait value of a population from one stage to the next.

The Washington State University Apple Breeding Program (WABP) Phase 2 field trials were used as a model to explore the influence of trial design factors on accuracy and cost. Under the current Phase 2 design, fruit from candidate selections are harvested three times per year per location (an attempt to harvest at optimum maturity) for a minimum of 3 years at three locations. Fruits are evaluated at harvest and after 8 weeks in 2°C regular atmosphere (RA) storage.^4^ The trial factors under consideration are the number of harvests per year, the number of years and the number of locations; the three criteria discussed above will be used to evaluate accuracy.

In-depth analysis of the genetic architecture of appearance and quality traits evaluated in the WABP was undertaken for the 2-month storage data used in this analysis and data from harvest assessments.^18^ Substantial interactions between candidates and locations, years or harvests was lacking for most traits, indicating a less-intensive assessment design could be used to predict candidate performance.^18^ Substantial candidate by storage duration interactions were lacking for all traits except firmness, indicating that selection could be made at either harvest or following 2 months storage.^18^ Therefore, short-term storage was recommended and chosen for this analysis as commercially sold apples are stored, thus data from that regime better reflects the commercial process.^18^

The aim of this study was to compare methods for evaluating trial design accuracy and cost in tree fruit and horticultural crop breeding, modeled using an operational apple breeding program. Other trial design accuracy and cost goals could be investigated with the outlined methods, however, this analysis compared alternative trial designs under which the total program cost remains constant. However, more candidates could be evaluated in each Phase 2 trial.

## Methods

### Current WABP Phase 2 trial design

Phase 2 is the most intensive data collection phase of the WABP and consists of replicated trials planted with promising selections from Phase 1.^18^ Phase 2 plantings under the current design are sited at three locations distributed across the major growing regions of Washington State each year. Five clonally propagated trees per candidate and several standard commercial cultivars are planted at each location in non-contiguous plots. Fruits harvested from the five clonal replicates are bulked into one sample; therefore there is no replication of a candidate within location. Plantings last no less than 4 years as most selections do not produce sufficient fruits to evaluate in the first year. Therefore, fruits from candidates can be considered to have been evaluated for 3 years.

Data collected included yield efficiency (not shown), fruit quality at harvest and after 8 weeks in 2 °C RA storage. Optimal harvest maturity was difficult to ascertain in new apple selections and influences important fruit quality traits—storability, flavor and texture. Selections were harvested three times at weekly intervals each year when fruits were mature. To determine the first harvest, maturity was estimated using the Cornell starch iodine index,^19^ and appearance. Fifteen fruits were sampled for each harvest; five fruits for instrumental analysis at harvest (not reported), and five fruits each for instrumental and sensory analysis after 8 weeks in storage. At the third harvest, all remaining fruits were harvested (that is, ‘strip-picked’) for other breeding program uses (not reported).

Sixteen ordinal traits were scored post storage (Appendix 1).^18^ Traits were evaluated by four experienced members of the breeding team, each trained in sensory analysis. Each member tasted and scored the apple sample; the average score was recorded. Sensory evaluations were based on anticipation of the consumer’s sensory perceptions. The evaluators’ perception of taste was assumed to be well correlated and representative of the consumer.

Six instrumental traits were also assessed after 8 weeks storage: SSC (soluble solids content, Brix, using a digital refractometer (RX-5000α-Bev, ATAGO USA, Inc., Bellevue, WA, USA); TA (titratable acidity, mg L^−1^ malic acid, using an auto-titrator (Metrohm 815 Robotic USB Sample Processor XL, Metrohm USA, Inc., Riverview, FL, USA); FRTDM (fruit diameter, inches), FRTWT (fruit weight, grams), M1 (firmness; lb) and CN (crispness; all assessed using a Mohr Digitest texture analyzer (MDT-1, Mohr Test and Measurement LLC, Richland, WA, USA^20^). Five apples were individually assessed using the texture analyzer. Fruits were then quartered; one quarter from the shoulder side of each were pooled and juiced. Fresh juice was then measured for SSC and remaining juice was frozen for later TA assessment. TA was not typically measured in real time due to the large number of samples being analyzed and subsequent time constraints.

### Cost assessment of the WABP

Current trial design costs were summarized in a bioeconomic spreadsheet model to examine the effect of alternative designs on trial costs (Table 1). The number of locations, years and harvests per year were varied, which changed the number of trees and fruit samples evaluated and therefore the associated field, consumable and labor costs. Locations were assumed to be equidistant and the average per site cost was used. Costs were divided into tree production, field establishment, field maintenance and candidate assessment, and presented on a per candidate basis. Cost of evaluating an individual was expressed on a scale where 100 units represented the total cost of evaluation of a single candidate in the current trial. This analysis was for the 4-year life of a single Phase 2 planting. Total per candidate cost (100 units) multiplied by the number of candidates typically evaluated in each Phase 2 trial (10) equalled the total program cost (1000 units) for the current design. To calculate the total number of individuals that could be evaluated under alternative designs, the total program cost was divided by the per candidate costs of each alternative design and rounded to the nearest whole number (Table 1). Design details were abbreviated so 3 locations, 3 years, 3 harvests per year is denoted 3L/3Y/3H. Alterations from the current design are in bold (that is, **2L**/3Y/3H). Designs considered were: single-factor alterations (**2L**/3Y/3H, 3L/**2Y**/3H, 3L/3Y/**2H**, 3L/3Y/**1H**); two-factor alterations (**2L**/3Y/**2H**, **2L**/**2Y**/3H, 3L/**2Y**/**2H)**; and **3L**/**2Y**/**2H**.

### Statistical methods

Variance components required for estimation of CPD, RS and CRS were estimated from a subset of data using 77 candidates evaluated between 2004 and 2011 that included only the 8-week storage assessment following methodology outlined by Hardner *et al.*^18^ with storage duration and relevant interaction terms removed, the final estimable model for individual trait observations presented was:

$$y=L*\left(Y+A\right)*H+G/\left(Y*H\right)+L^{ˆ}P/\left(Y*H\right)+e$$

where *L* is location (F, Farm in Hardner *et al.*^18^), *Y* is year (S, Season in Hardner *et al.*^18^), *A* is age, *H* is harvest, *G* is candidate and *P* is plot. The symbol ^ denotes the interaction between terms, / denotes nesting of terms (that is, *A*/*B*=*A*+*A*^*B*) and * implied a full expansion of terms (that is, A*B=*A*+*B*+*A*^*B*). Terms from the expansion of L* (*Y* + *A*)*H were treated as fixed and the rest as random.

The general mixed linear random model used to estimate variance components and test fixed effects for fruit quality traits assessed after 2 months of storage was the same as used by Hardner *et al.*^18^ Variance components for the random factors defined in the mixed model were estimated by Restricted Maximum Likelihood^21^ with the software ASReml.^22^ The Shapiro–Wilk statistic^23^ was calculated for the residuals to examine the assumption of normality (not shown). Wald statistics were used to test fixed effects.^24^ The likelihood ratio test was used to test significance of random terms^25^ with *P*=0.05.

For a balanced trial design where candidates (*G*) were harvested (*H*) *h* times at *l* locations (*L*) in each *y* years (*Y*), total variance of the predicted candidate effect (*σ*^*2*^_*Ĝ*_) was given by:

$${\sigma^{2}}_{\hat{G}}=\frac{{\sigma^{2}}_{GL}}{l}+\frac{{\sigma^{2}}_{GY}}{y}+\frac{{\sigma^{2}}_{GH}}{h}+\frac{{\sigma^{2}}_{GLY}}{ly}+\frac{{\sigma^{2}}_{GLH}}{lh}+\frac{{\sigma^{2}}_{GYH}}{yh}+\frac{{\sigma^{2}}_{GLYH}}{lyh}$$

Plot variance was confounded with location variance as there were no plots within location; if *G*×plot variance was assumed to be zero, then the total variance due to differences in trial locations was in the *G*×*L* variance. Year variance was confounded with candidate age variance. If candidate age variance was assumed to be zero, then the total differences in the trial years was in the *G*×*Y* variance. Total genetic variation was calculated using methods outlined by Hardner *et al.*^18^

### Critical Percentage Difference

CPD, the difference in the sample mean needed to reject the null hypothesis with a level of confidence of *α*, was estimated as:

$$CPD\left(\alpha \right)=\frac{100*2\sqrt{\sigma_{\hat{G}}^{2}}}{µ}Z_{\alpha /2}$$

where σGx^2 was the s.e. of the predicted candidate effect (described above) and the standard normal distribution value for *α*=0.05 was *Z*_*α/2*_=1.96.^13^ The null hypothesis was that two entries have an equal true mean. The two-tailed hypothesis was chosen because selections entered Phase 2 trials to test their performance against standards, and there is uncertainty as to whether they are better or worse than the standards for a particular trait before the results. CPD was presented for the current design. To allow for comparisons between traits and designs, differences in CPD between an alternative design and the current design were presented. A positive change in CPD for an alternative design indicated that the alternative design was less accurate than the current design at predicting candidate effect.

To determine if the effect of a combined change in multiple factors was different from the sum of the effects of independent changes in each factor (that is, **2L**/3Y/3H versus **2L**/**2Y**/3H), the difference between the single-factor altered design and the current design was calculated and those differences were added to get an expected CPD. A difference between actual and expected CPD indicated the presence of this type of interaction.

### Response to Selection

RS for a balanced design was given as:

RSx=iτGˆx,Gxσ2Gx^+σG2

where the selection intensity (*i*) equals 1.76 if the proportion of individuals selected was 0.1,^6^ *τ*_*Ĝ*x,*G*x_ was the covariance between the predicted candidate effect and the true candidate effect, and σGx^2 was the s.e. of the predicted candidate effect. This equation was derived from Falconer and Mackay (Appendix 2).^6^ The selection intensity chosen represented the current selection intensity (1 candidate advanced from a pool of 10) in the WABP. Changes in RS were either from selection intensity or variation in the predicted candidate effect. By designating the selection intensity, RS reflected changes in variation of predicted candidate effect due to changes in trial designs.

To determine if the effect of a combined change in multiple factors was different from the sum of the effects of the independent changes in each of the factors (that is, **2L**/3Y/3H versus **2L**/**2Y**/3H), the difference between the single-factor altered design and the current design was calculated and those differences were added to get an expected RS. An interaction was indicated by a difference between actual and expected RS, as above for CPD.

### Correlated response

CRS was calculated for traits measured both instrumentally and organoleptically: CN v. CRISP; M1 v. HARD; SSC v. SWEET; TA v. TART. The underlying assumption was that the WABP’s sensory evaluation of traits approximates the average consumer’s assessment. To explore how selection for an instrumental trait (*y*) changed the response of a second, correlated sensory trait (*x*) under various trial designs, correlated response for a balanced design was calculated as:

$$CRS_{Gy}=i\frac{rG_{x}G_{y}\sqrt{{\sigma^{2}}_{G_{x}}{\sigma^{2}}_{{\hat{G}}_{y}}}}{\sqrt{{\sigma^{2}}_{{\hat{G}}_{y}}}}$$

where *rG_x_G_y_* is the genetic correlation between trait *x* and *y*, *σ^2^_Gx_* is the variance of the genetic effect of trait *x* and *σ^2^_Ĝy_* is the variance of the predicted candidate effect of trait *y*. Selection intensity (*i*) equalled 1.76 when the proportion of individuals selected was 0.1 and 2.06 when the proportion was 0.05. This equation was derived from Falconer and MacKay (Appendix 2).^6^ Other parameters were assumed constant therefore changes in CRS were due to variation in selection intensity or variation in predicted candidate effect. Changes in CRS were the effect of trial design changes on the variation in predicted candidate effect of the sensory trait when selection intensity was invariant.

Genetic correlations estimated from a previous analysis of fruit quality traits after storage for entries in existing WABP field trials ^18^ were used to estimate correlated response (Appendix 3). Correlated responses were in units of trait targeted by selection, which were the ordinal sensory traits CRISP, HARD, SWEET, and TART.

## Results

### Variance components

Genetic effects accounted for 18–65% of phenotypic variance for fruit quality traits and the residuals were normally distributed (Table 2). The main effect of candidate was the largest source of phenotypic variance for all appearance traits except APPSUM, GCOL, RUSS and SHAPE, all instrumental traits except CN and SSC, but only TART of the sensory traits (Table 2). For those traits, residuals accounted for the largest source of variance. *G*×*H* interaction was zero for all traits except OVERALL. Variation due to *G*×*L* was significant for all traits except CN and TART, and there was significant variation due to *G*×*Y* for all except M1 and TART. *G*×*L* was smaller or equal to *G*×*Y* except for SIZE, M1 and TA. Few significant interactions were detected for *G*×*Y*×*H* (TCOL) or *G*×*L*×*H* (GCOL, TA). There was significant variation for *G*×*L*×*Y* for all traits except OVERALL.

### Cost

Candidate assessment was the most expensive component of the current design, accounting for 74.2 of the total 100 units (Table 1). Costs incurred during candidate assessment included staff time required for driving, 27 harvests (three locations harvested nine times over 3 years), data collection and data entry, as well as fuel, vehicle and consumable expenses. Driving costs were the most expensive sub-component of candidate assessment, accounting for 50.7 units. Data collection was the second most expensive sub-component of candidate assessment, accounting for 15.5 units. Sensory analysis was the most expensive sub-component of data collection and the third most expensive individual component of the whole trial.

#### Changes in costs

For the single-factor altered designs, the largest cost reduction was seen under the **2L**/3Y/3H design. Decreased tree production, field establishment, field maintenance and candidate assessment resulted in per candidate cost reduction of 33.3 units; therefore, five additional candidates could be evaluated for the same total program cost (Table 1). Reduction in per candidate costs was smallest under the 3L/3Y/**1H** design due to tree production, field establishment, maintenance and driving costs remaining the same as the current design (Table 1). Despite the decrease in harvest number, driving costs would not decrease as field trials would still need to be visited each week due to the number of candidates and their varying ripening date. Only two additional candidates could be evaluated for the same total program cost.

Reducing two factors simultaneously resulted in greater reductions in per candidate cost than reducing only one factor, except in the case of 3L/**2Y**/**2H** which was 2.6 units more than **2L**/3Y/3H. The **2L**/**2Y**/3H design resulted in the greatest reduction in per candidate costs, by 50.8 units, due to the reduced number of strip-picks (four, six for both **2L**/3Y/**2H**, and 3L/**2Y**/**2H**; Table 1). Ten additional candidates could be evaluated for the same total program cost. Reducing all three factors simultaneously resulted in the greatest reduction in per candidate cost and 12 additional candidates could be evaluated.

### Effect on accuracy

#### Changes in accuracy from altering one design factor

Accuracy was reduced, as measured by CPD, for all single-factor altered designs as there were less observational units; however, reduction in accuracy was <5% for most traits (Table 3). The smallest decrease in accuracy was under the 3L/3Y/**2H** design for all traits. The only decreases in accuracy >5% were under the 3L/3Y/**1H** design: OVERALL, CN, GCOL, SHAPE and TCOL.

#### Changes in accuracy from altering two design factors

Altering two factors simultaneously resulted in greater reduction in accuracy than altering a single factor. This reduction was again <5% for most traits (Table 3).

For the sensory traits CRISP, HARD, JUIC, AROM, TART and EQ, the smallest decrease in accuracy was under both **2L**/3Y/**2H** and **2L**/**2Y**/3H. The reduction in accuracy for OVERALL (6) and SWEET (2.7) were equal under all three designs.

For instrumental traits, the decrease in accuracy for CN was >5% for all three designs, with the smallest reduction under the **2L/**3Y**/2H** design. The smallest decrease in accuracy for M1 and TA was under 3L**/2Y/2H**. The smallest decrease in accuracy for SSC and FRTDM were under both **2L**/3Y/**2H** and **2L**/**2Y**/3H. The decrease in accuracy for FRTWT (4.1) was equal for all two-factor altered designs.

For appearance traits, CPD values were equal under both 3L/**2Y**/**2H** and **2L**/3Y/**2H** for for PCOL (3.8). The decrease in accuracy under both **2L**/3Y/**2H** and **2L/2Y/**3H designs for APPSUM, GCOL, LENT, RUSS, SHAPE and TCOL were similarly small. The smallest decrease in accuracy for SIZE was under the 3L/**2Y**/**2H** design. The decrease in accuracy was >5% for GCOL, RUSS and SHAPE under 3L/**2Y**/**2H**, and for TCOL under all three designs.

#### Changes in accuracy from altering three design factors

Decreases in accuracy were >5% for most traits under **2L**/**2Y**/**2H**. The greatest reduction in accuracy was for CN (11.8) and OVERALL (10.1).

#### Interactions

CPD values for two- or three-factor altered designs were generally higher than expected based on the sum of CPD values for single-factor altered designs. FRTWT, PCOL and SIZE are the exceptions, having lower than expected CPD values for the 3L/**2Y/2H**, **2L/2Y**/3H and **2L/2Y/2H** designs. Differences between expected CPD values and actual CPD values were generally <1%. CPD values were higher than the expected 2% for GCOL, LENT and RUSS with 3L/**2Y/2H**, **2L/2Y**/3H and **2L/2Y/2H**.

CPD values were also higher than the expected 1% for AROM, OVERALL CN, SHAPE and TCOL for **2L/2Y/2H**, which indicated an interaction between the one factor alterations and this design.

### Effect on RS

#### Changes in RS from altering one design factors

For most traits, there was <5% reduction in RS under any single-factor altered design (Table 4) with almost no reduction for any trait under 3L/3Y/**2H**. The only reductions larger than 5% were seen under 3L/3Y/**1H**, with the largest seen for SWEET (13%).

#### Changes in RS from altering two design factors

As seen for CPD, there was <5% reduction in RS for most traits under two-factor altered designs (Table 4). For sensory traits, reductions in RS were larger than 5% for AROM, OVERALL and SWEET for all three two-factor altered designs as well as for EQ under **2Y**/**2L**/3H and JUIC under both 3L/**2Y**/**2H** and **2L**/**2Y**/3H. The largest decrease in RS for most traits was under **2L**/**2Y**/3H. For instrumental traits, there was a 5% or larger decrease in response for CN under **2L**/**2Y**/3H and **2Y**/**2L**/3H, and for both SSC and TA under all two-factor altered designs. For appearance traits, few reductions in RS were equal to 5% and none were larger.

#### Changes in RS from altering three design factors

The largest reduction in RS for most traits was under **2L**/**2Y**/**2H** (Table 4). The largest reduction in RS, as with CPD, was for OVERALL (13%) and SWEET (13%).

#### Interactions

As with CPD, RS values for two- or three-factor altered designs were higher than expected for most traits based on the sum of RS values for single-factor altered designs. However, the reduction in response for most traits under the two-factor altered designs were generally small.

For the three-factor altered design, RS values were greater than expected for all sensory and instrumental traits, except for JUIC where RS values equaled expected. For appearance traits, the decrease in RS was equal to expected for TCOL and smaller than expected for APPSUM.

### Correlated response

For the current design (3Y/3L/3H), direct selection on sensory CRISP increased the selected candidates’ trait average by 0.63 units on the ordinal scale, while indirect selection increased the average by 0.5 units (Table 4). Indirect selection to improve CRISP was less effective than direct selection for all designs considered. Similarly, direct selection on SWEET resulted in greater gains than indirect selection on SSC for all designs. Indirect selection on M1 made slightly greater progress or equaled direct selection for all considered designs. Selecting indirectly on TA and directly on TART both resulted in a gain of 0.56 for the current design, **2L**/3Y/3H and 3L/3Y/**2H**. Indirect selection using TA was slightly advantageous for the other designs.

### Changing intensity

Increasing selection intensity by reducing the proportion of selected candidates increased RS values for all traits as well as CRS values for all trait pairs (Table 4). Under the current design, where one out of 10 candidates (10%) were selected, cost per candidate was 100 units with a total program cost of 1000 units (100×10; Table 1). The smallest reduction in accuracy and RS for most traits was under the **2L**/3Y/**2H** design. For the current total program cost, six additional candidates could be evaluated. RS was highest at the 5% selection intensity for all traits and if ~6% of candidates were advanced (1 out of 16) with a per candidate cost of 62.2 units, then greater progress could be made in the average trait values from Phase 2 to the next phase. CRS of all trait pairs increased; however, HARD and TART remained the only sensory traits that benefit more from indirect selection.

## Discussion

There are inherent trade-offs between accuracy and cost in trial design efficiency. Using costs and data from an operational apple breeding program as a model, the ramifications of reducing the number of levels of one or more factors in a trial design for advanced breeding material was explored in order to evaluate additional candidates. The total cost of the program could be reduced if the trial design were less intensive (that is, number of years, locations or harvests per year were reduced), but this leads to a decrease in accuracy and RS. Cost savings, from a reduced trial design, that allow evaluation of additional candidates warrant the decreases in accuracy and RS for the program examined. This study demonstrates the applicability of these methods to tree fruit and horticultural crop breeding and the utility of the results in making informed trial design decisions.

### Variance of fruit quality traits

More than half of the traits included in this analysis have residual variances as the largest single source of variance. Residual variances were greater than those reported by Hardner *et al.*^18^ partially due to the removal of storage duration variance components. This could be due to a lack of consistency between observations not attributable to year, location, harvest and their interactions, or the inherent variability among fruit for these traits.^18^

Summary traits APPSUM, EQ and OVERALL had relatively high residual variances, with OVERALL having the largest residual variance of all of the traits. Other factors influence scoring decisions for summary traits, and those factors are not necessarily consistent between candidates. Consider two equally attractive apple samples evaluated by the breeding team, where one sample had large, bulbous stems that may increase water loss and the other had open calyxes that may increase susceptibility to core rots. Both samples would receive the same lower APPSUM rating despite similar marks on the other ordinal appearance traits. OVERALL, in particular, is influenced by many unscored factors as this ‘trait’ denotes selection decision. The three anchors for OVERALL on the ordinal scale are ‘Reject’, ‘Re-evaluate’ or ‘Advance’. A fruit sample could be scored as ‘reject’ for a number of reasons not covered in any of the other 15 traits or for post-harvest disorder incidence scores (not reported). Overall perception of a fresh-eaten apple is the interaction between the numerous traits measured by the WABP and the summary traits serve as a useful way to rate those interactions.

### Performance of trial designs

Reducing one factor of the design resulted in small reductions in accuracy for both CPD and RS, but less reduction in cost. There was a greater reduction in accuracy when reducing two factors simultaneously, as well as greater reductions in cost. CPD and RS values that were greater or smaller than expected for two- and three-factor altered designs indicated that there were interactions. These reflected interactions between candidate and random effects (that is, *G*×*L*, *G*×*Y*, *G*×*Y*×*L*), highlighting the importance of analyzing the genetic architecture of traits before this type of analysis.

Reducing both harvests and locations (**2L**/3Y/**2H**) resulted in the smallest decrease in accuracy of the two-factor altered designs and would allow the program to evaluate 12 additional candidates for a similar total program cost. Interactions for *G*×*H*, *G*×*H*×*Y* and *G*×*H*×*L* were very small or non-existent for all of the traits evaluated, which may explain the <5% reduction in accuracy and gain for most traits. Decrease in accuracy and RS for some of the most important traits for advancement decisions (CRISP, JUIC, TART, EQ and OVERALL) were <5%. A lack of *G*×*L* in WABP Phase 2 trials^18^ suggests that central Washington could be considered one selection environment. Removing a location resulted in negligible decreases in accuracy and gain, but sizeable savings of 33.3 units per candidate cost. This suggests there may be a little value in the third location in terms of improving accuracy and RS. **2L**/**2Y**/3H was the other two-factor altered design with relatively small decreases in both accuracy and RS, and larger reductions in cost than **2L**/3Y/**2H**. *G*×*Y* and *G*×*L*×*Y* interactions were large for most traits. Under the current design, *G*×Age variance is confounded with *G*×*Y* variance. Our experience indicates that the initial crop(s) on young trees may not accurately represent a candidate selection’s fruit quality. Further studies could be conducted with the same genetic material planted over multiple years. Until then, the 2 years of fruit evaluations could be the third and fourth crop of the Phase 2 trials, while still reducing assessment costs by 41.3 units from the current design.

The presented accuracy and RS estimates assume that each trial is successful. Freezes that damage buds (fall), blossoms (spring), young fruitlets (spring) or hail damage on fruit (summer) can compromise an entire year’s data which would delay advancement decisions. In a crop such as apple, where time to release is already close to 20 years, each year of delay sacrifices speed and incurs considerable costs. The same risk management concerns for reducing years applies to reducing locations, especially if fruit assessment was delayed to the last 2 years. For that reason, reducing all factors (**2L**/**2Y**/**2H**) or both locations and years (**2L**/**2Y**/3H) seemed too high risk for the WABP.

The effect of reducing the number of trees per candidate per location could also have been considered in this analysis; however, that option seemed too high risk for this program. Five trees are needed to ensure sufficient yield for evaluation and blossoms for use as parents, particularly as some trees fail to thrive or die outright due to field variation or irrigation issues.

Furthermore, stakeholder interests must be included. Growers are the ultimate consumer of new releases. Removing a site near a large proportion of interested growers or that is politically important may decrease their confidence and support in the program. All crop breeding programs are susceptible to pernicious weather and serve their stakeholders to varying degrees, and thus must consider these contingencies.

### Considerations for implementing new design in an operational breeding program

Trial design accuracy must be balanced with managing risk, particularly in tree fruit crops where the investments in breeding and by the grower in planting a new variety, are very high. An alternative option that further reduces risk of **2L**/3Y/**2H** may be to plant an incomplete block, where each candidate was randomly planted at two of three locations. This reduces propagation, field establishment and maintenance costs. Conversely, a complete block could be planted, but only two of the three locations would be evaluated each year, saving much of the assessment costs associated with driving to harvest, harvesting, evaluating fruit and data entry. The third site acts as insurance in the event that one location is compromised (that is, freeze, hail and so on). Cost for a single candidate in this scenario is 70.8 units. For a total program cost of 991 units, four additional candidates could be evaluated (14 total). CPD values for this design would be the same as the **2L**/3Y/**2H** design. RS and CRS values would be between the 10 and 5% proportion selected values presented, but more complex methods would be needed to evaluate this quantitatively. Intensifying selection, or increasing the number of candidates evaluated without increasing the number of candidates advanced to the next phase, results in greater gains in trait values. The subsequent increase in RS values indicates that overall quality of candidates advanced to the next phase would likewise increase.

### Utility of correlated response to selection in trial design considerations

Sensory evaluations tend to be expensive to measure, have lower heritabilities due to high variability^17^ and suffer from ‘taste-fatigue’,^16^ which was the motivation for investigating indirect selection using instrumental measures. Tartness (TART, TA), sweetness (SWEET, SSC), crispness (CRISP, CN) and firmness (HARD, M1) are measured both organoleptically and instrumentally in the WABP. CRS indicated that more gain could be made for crispness and sweetness using direct selection of the sensory trait while tartness and firmness benefited from indirect selection using the instrumental measure. Genetic correlations for both SWEET and SSC (0.57), and CRISP and CN (0.75) were lower than for firmness or tartness (Appendix 4). Heritabilities were higher for TA, M1 and CRISP than their paired trait and gained more from direct selection (Table 2). Heritability of SSC was higher than for SWEET (0.24, 0.21); however, direct selection on SWEET made greater progress. This seeming discrepancy could be due to the low heritability of both measures of sweetness, the significant interactions between *G*×*Y* and *G*×*L*×*Y* observed for SSC or that SWEET is determined by genetic factors other than SSC.

Sensory analysis is an expensive component of fruit assessment, and the results of this study suggest that organoleptic scoring of TART and HARD could be removed. However, removing them would not decrease costs as both traits are scored with the other organoleptic traits and subjective sensory analysis ultimately drives selection decisions.^3,26^

A similar argument could be made to remove the instrumental measurements CN and SSC to reduce costs. CN is measured as part of a suite of variables on the Mohr Digitest, so removing CN would not reduce assessment costs. However, SSC adds to both assessment time and data collation. Nurserymen and growers are interested in quantitative SSC values at harvest and after storage to facilitate objective comparisons between cultivars and potential selections (that is, ref. 27), as well as an indication of harvest maturity.^28^ SSC measurements could be restricted to Phase 3 as a way to reduce costs of candidate assessment. Although machine scoring offered little advantage in this program where organoleptic scoring is performed by a trained panel, it may offer greater benefits to those programs utilizing an un-trained or less discerning panel.

RS and CRS assume that the type of selection for the trait is directional (that is, increasing or decreasing). Trial averages for several important traits (that is, TART, HARD) are within the desired range; directional progress for those traits would produce overly tart or low acid, and very firm or very soft apples, thus stabilizing selection is the goal. RS and CRS values may not be directly applicable for those traits, but are useful in understanding accuracy for either type of selection. Irrespective of directional or stabilizing selection, RS and CRS for these traits is almost as accurate under the reduced designs as they are under the current design.

## Conclusion

Methods employed in this analysis offer a framework for other tree fruit and horticultural crop breeding programs to investigate their unique trial design accuracy and efficiency questions. Considerations specific to the WABP were outlined to demonstrate that trial design accuracy and cost must also be considered with program-specific needs, including risk management, stakeholder needs and characteristics of individual traits. All breeding programs face challenges that would similarly inform their interpretation and the utility of the results.

Programs may want to examine improving accuracy while keeping program cost static by reducing some factors and increasing other factors. Additional methodologies could be used to investigate the effect of unbalanced trial designs.^29^

Previous analyses similar to this were undertaken in agronomic crops, where increasing yield was the main consideration and directional selection is employed.^9,12,13,30^ Dessert apples are somewhat unique in that multiple flavor and texture profiles are acceptable and expected by consumers,^31,32^ and thus breeding targets are equally diverse. Despite utilization of this multi-targeted selection, the analysis yielded valuable results. The utility of the trial design efficiency analysis outlined in this study indicates that it would be equally, if not more, useful for breeders of other tree fruit and horticultural crops.

## Figures and Tables

**Table 1:** Phase 2 trial component costs for a single candidate standardized to the current design, total standardized Phase 2 trial cost and the total number of candidates that could be evaluated for the current total WSU apple breeding program cost for the current Phase 2 trial design and alternative trial designs

	*Current: 3 Locations, 3 Years, 3 Harvests*	***2 Locations**, 3 Years, 3 Harvests*	*3 Locations, **2** Years, 3 Harvests*	*3 Locations, 3 Years, 1 Ha**rvest***	*3 Locations, 3 Years, 2 H**arvest***	*3 Locations, **2 Years, 2 Harvests***	***2 Locations**, 3 Years, 2 Harvests*	***2 Locations, 2** Years, 3 Harvests*	*2 Locations, 2 Years, 2 Harvest**s***
Nursery production of trees	7.0	4.7	7.0	7.0	7.0	7.0	4.7	4.7	4.7
Field establishment^a^	12.9	8.6	12.9	12.9	12.9	12.9	8.6	8.6	8.6
Field maintenance^b^	5.7	3.8	4.3	5.7	5.7	4.3	3.8	2.9	2.9
									
*Candidate assessment*									
Driving^c^	50.7	33.8	33.8	50.7	50.7	33.8	33.8	22.5	22.5
Harvest	6.7	4.5	4.5	4.2	5.5	3.6	3.6	2.9	2.4
Data entry	1.6	1.1	1.1	0.5	1.1	0.7	0.7	0.7	0.5
Consumables^d^	0.1	0.1	0.1	0.1	0.1	0.1	0.1	0.0	0.0
Data collection									
Tree diameter	0.4	0.2	0.2	0.4	0.4	0.2	0.2	0.2	0.2
Sensory analysis	10.1	6.7	6.7	3.4	6.7	4.5	4.5	4.5	3.0
Instrumental analysis	4.8	3.2	3.2	1.6	3.2	2.2	2.2	2.2	1.4
Total cost (units) per candidate	100.0	66.7	73.8	86.5	93.3	69.3	62.2	49.2	46.2
Total program cost (unit cost per candidate x 0 candidates)	1000	667	738	865	933	693	622	492	462
Total candidates that could be evaluated for 1000 units	10	15	14	12	11	14	16	20	22

Actual costs were converted to units such that 100 units represents the total costs of evaluation of a single candidate in the current design.

a Includes fumigation, trellis materials, planting time and labeling costs.

b Includes plot fees (trellis installation, pesticide application and irrigation), pesticides, pruning, thinning and orchard removal after 4 years.

c Includes staff time and per mile reimbursement for 12 weeks of the harvest season.

d Includes labels printed for each sample.

**Table 2:** Estimated phenotypic variance (v.P), percentage of estimated variance components for individual random effects (G: candidate, H: harvest, L: Location, E: residual error), interactions from the analysis of individual traits, and total genetic variation (*H*^2^) for the fruit quality traits assessed as part of the WABP Phase 2 trials

*%*										
*Trait*	*v.P*	*G*	*G.Y*	*G.H*	*G.Y.H*	*G.L*	*G.L.Y*	*G.L.H*	*E*	*H*^*2*^
*Sensory*										
AROM^a^	0.46	26	7	0	0	3	16	0	**48**^b^	0.26
CRISP	0.34	42	4	0	0	2	5	0	**47**	0.42
EQ	1.04	30	4	0	0	5	6	0	**55**	0.30
HARD	0.32	38	6	0	0	1	4	0	**51**	0.38
JUIC	0.26	27	8	0	0	2	6	0	**57**	0.27
OVERALL	0.23	18	4	2	0	4	0	0	**72**	0.18
SWEET	0.23	21	8	0	0	8	7	0	**56**	0.21
TART	0.24	**46**	0	0	4	0	8	0	42	0.46
										
*Instrumental*										
CN	4447	30	5	0	0	0	10	0	**55**	0.31
FRTDM	0.08	**51**	5	0	0	4	11	0	29	0.51
FRTWT	3498	**49**	5	0	0	5	11	0	30	0.49
M1	6.42	**61**	0	0	0	2	16	0	21	0.61
SSC	1.18	24	11	0	0	5	13	0	**47**	0.24
TA	0.02	**57**	3	0	0	4	2	2	32	0.57
										
*Appearance*										
APPSUM	0.38	38	7	0	0	4	6	0	**45**	0.38
GCOL	0.33	38	8	0	0	1	7	3	**43**	0.38
LENT	0.58	**52**	6	0	0	2	5	0	35	0.52
PCOL	1.43	**65**	4	0	0	4	2	0	25	0.65
RUSS	0.96	37	8	0	0	2	8	0	**45**	0.37
SHAPE	0.91	38	8	0	0	5	0	0	**49**	0.39
SIZE	0.50	**43**	4	0	0	5	9	0	39	0.43
TCOL	0.45	**59**	3	0	2	2	6	0	28	0.59

Zero variance component indicates source of variation was not significant.

a Additional details for appearance and sensory traits given in Appendix 1.

b The single largest source of variance for each trait is emboldened.

**Table 3:** Critical percentage difference required between sample means to reject the hypothesis that two candidates have the same true mean with 95% confidence for traits assessed during Phase 2 trials by the WABP

	*%*		*Current %–Alternative %*							
	*Current trait average*	*Current: 3 Locations, 3 Years, 3 Harvests*	***2 Locations**, 3 Years, 3 Harvests*	*3 Locations, **2 Years**, 3 Harvests*	*3 Locations, 3 Years, **1 Harvest***	*3 Locations, 3 Years, **2 Harvest***	*3 Locations, **2 Years, 2 Harvests***	***2 Locations**, 3 Years, **2 Harvests***	***2 Locations, 2 Years**, 3 Harvests*	***2 Locations, 2 Years, 2 Harvests***
AROM	2.9	17	2.6	3.3	3.9	1.1	4.6	4	4	8.1
CRISP	3.18	10.5	1.7	2	3.6	1	3.3	3	3	5.6
EQ	5.78	11.7	2.1	1.9	3.6	1	3.2	3.3	3.3	5.9
HARD	3.54	9.6	1.3	2	3.3	0.9	3.2	2.5	2.5	5.1
JUIC	3.4	10.2	1.4	2.1	3.1	0.9	3.1	2.5	2.5	5
OVERALL	1.7	19.2	3	3	8.7	2.5	6	6	6	10.1
SWEET	3.28	11.5	1.8	1.8	2.7	0.7	2.7	2.7	2.7	5
TART	3.32	6.9	1.3	1.6	3.8	1.1	2.9	2.6	2.6	4.8
CN	195.5	20.8	3.2	4.7	7.4	2.1	7.2	5.9	8.5	11.8
FRTDM	2.94	6.2	1	1.1	1.2	0.3	1.5	1.4	1.4	2.7
FRTWT	209.54	18.6	3	3.9	3.3	0.9	4.1	4.1	4.1	7.8
M1	18.42	6.9	1.6	1.3	1.6	0.4	1.8	2.1	2.1	3.7
SSC	13.68	6.4	0.9	1.2	1.2	0.3	1.6	1.3	1.3	2.7
TA	0.64	12	2.1	1.7	3.7	1	3	3.4	3.4	5.5
APPSUM	2.92	13.5	1.9	2.4	3.3	0.9	3.6	3.1	3.1	6.1
GCOL	2.04	18.7	2.3	2.9	5.4	1.5	5.5	4.3	4.3	8.8
LENT	3.04	14.8	1.9	1.4	3.8	1	4.2	3.3	3.3	6.8
PCOL	3.86	16.7	2.5	4.1	3.6	1	3.8	3.8	3.8	6.8
RUSS	3.82	17.3	2.2	1.9	4.3	1.2	5	3.8	3.8	8
SHAPE	2.98	22	2.9	3.7	5.7	1.5	5.7	4.9	4.9	9.5
SIZE	3.26	14	2.4	3.7	3.2	0.9	3.3	3.5	3.5	6.4
TCOL	1.78	20	3.1	3.7	6.2	1.7	5.9	5.2	5.2	9.9

Trait averages are presented for the current design in the unit of the trait as is the CPD as a percentage. CPD presented for alternative designs were subtracted from the current design to give the degree of change rather than absolute value.

**Table 4:** Response to selection for traits assessed during Phase 2 trials by the WABP and correlated response to selection for four selected pairs of traits (in italics) under the current and alternative trial designs

	*Current: 3 Locations, 3 Years, 3 Harvests*	***2 Locations**, 3 Years, 3 Harvests (%)*	*3 Locations, **2 Years**, 3 Harvests (%)*	*3 Locations, 3 Years, **1 Harvest** (%)*	*3 Locations, 3 Years, **2 Harvest** (%)*	*3 Locations, **2 Years, 2 Harvests** (%)*	***2 Locations**, 3 Years, **2 Harvests** (%)*	***2 Locations**, **2 Years**, 3 Harvests (%)*	***2 Locations, 2 Years, 2 Harvests*** *(%)*	***2 Locations**, 3 Years **2 Harvests**, **5% SI** (%)*
AROM	0.54	0.52/−4	0.52/−4	0.51/−6	0.53/−2	0.51/−6	0.51/−6	0.5/−7	0.48/−11	0.6/+11
CRISP	0.63	0.62/−2	0.62/−2	0.61/−3	0.63/0	0.61/−3	0.62/−2	0.61/−3	0.60/−5	0.72/+14
EQ	0.9	0.87/−3	0.88/−2	0.85/−6	0.89/−1	0.86/−4	0.86/−4	0.84 /−7	0.82/−9	1/+11
HARD	0.58	0.57/−2	0.57/−2	0.56/−3	0.58/0	0.56/−3	0.56/−3	0.56/−3	0.54/−7	0.66/+14
JUIC	0.42	0.41/−2	0.4/−5	0.4/−5	0.41/−2	0.39/−7	0.4/−5	0.39/−7	0.38/−10	0.47/+12
OVERALL	0.31	0.3/−3	0.3/−3	0.27/−13	0.3/−3	0.29/−6	0.29/−6	0.28/−10	0.27/−13	0.34/+10
SWEET	0.32	0.31/−3	0.31/−3	0.3/−6	0.32/0	0.3/−6	0.3/−6	0.29/−9	0.28/−13	0.35/+9
TART	0.56	0.56/0	0.55/−2	0.54/−4	0.56/0	0.55/−2	0.55/−2	0.54/−4	0.53/−5	0.64/+14
CN	59.68	58.37/−2	57.72/−3	56.47/−5	58.83/−1	56.57/−5	57.19/−4	55.97/−6	54.42/−9	67.03/+12
*CR*_*CRISP*_	*0.5*	*0.5/0%*	*0.5/0%*	*0.5/0%*	*0.5/0%*	*0.5/0%*	*0.5/0%*	*0.5/0%*	*0.5/0%*	*0.58/+16%*
FRTDM	0.34	0.34/0	0.34/0	0.34/0	0.34/0	0.33/−3	0.33/−3	0.33/−3	0.33/−3	0.39/+15
FRTWT	69.01	67.79/−2	67.79/−2	67.69/−2	68.68/0	67.32/−2	67.32/−2	66.31/−4	65.65/−5	78.91/+14
M1	3.39	3.35/−1	3.36/−1	3.35/−1	3.38/0	3.34/−1	3.34/−1	3.3/−3	3.28/−3	3.91/+15
*CR*_*HARD*_	*0.59*	*0.59/0%*	*0.59/0%*	*0.59/0%*	*0.59/0%*	*0.59/0%*	*0.59/0%*	*0.59/0%*	*0.59/0%*	*0.69/+17%*
SSC	0.81	0.78/−4	0.77/−5	0.77/−5	0.8/−1	0.75/−7	0.76/−6	0.73/−10	0.72/−11	0.9/+11
*CR*_*SWEET*_	*0.22*	*0.22/0%*	*0.22/0%*	*0.22/0%*	*0.22/0%*	*0.22/0%*	*0.22/0%*	*0.22/0%*	*0.22/0%*	*0.26/+18%*
TA	0.18	0.18/0	0.18/0	0.17/−6	0.18/0	0.17/−6	0.17/−6	0.17/−6	0.17 /−6	0.2/+11
*CR*_*TART*_	*0.56*	*0.56/0%*	*0.56/0%*	*0.56/0%*	*0.56/0%*	*0.56/0%*	*0.56/0%*	*0.56/0%*	*0.56/0%*	*0.66/+18%*
APPSUM	0.59	0.57/−3	0.57/−3	0.57/−3	0.58/−2	0.56/−5	0.57/−3	0.56/−5	0.55/−7	0.66/+12
GCOL	0.58	0.58/0	0.57/−2	0.56/−3	0.58/0	0.56/−3	0.57/−2	0.56/−3	0.55/−5	0.66/+14
LENT	0.93	0.92/−1	0.91/−2	0.91/−2	0.93/0	0.91/−2	0.91/−2	0.9/−3	0.89/−4	1.07/+15
PCOL	1.65	1.64/−1	1.64/−1	1.63/−1	1.64/−1	1.63/−1	1.63/−1	1.62/−2	1.61/−2	1.91/+16
SHAPE	0.96	0.94/−2	0.94/−2	0.93/−3	0.95/−1	0.93/−3	0.93/−3	0.92/−4	0.9/−6	1.09/+14
SIZE	0.77	0.76/−1	0.76/−1	0.75/−3	0.77/0	0.75/−3	0.75/−3	0.74/−4	0.73/−5	0.88/+14
RUSS	0.97	0.96/−1	0.95/−2	0.94/−3	0.97/0	0.93/−4	0.94/−3	0.92/−5	0.91/−6	1.11/+14
TCOL	0.89	0.88/−1	0.87/−2	0.87/−2	0.88/−1	0.87/−2	0.87/−2	0.86/−3	0.85/−4	1.02/+15

Percentage change in response is presented; negative values indicate a decrease in response and positive values indicate an increase in response. Selection intensity (SI) is 10%, unless noted. RS is in the unit of the trait and CRS is in the unit of the sensory trait.

**Table 5 tbl5:** Descriptions of ordinal traits scored after 8 weeks in regular atmosphere 2 °C storage and 1 week shelf-life test at room temperature.

*Trait abbreviation*	*Trait description*	*Scale description*
*Ordinal appearance traits*		
APPSUM	Appearance summary	11 Increments from ugly to beautiful
GCOL	Predominant background (‘ground’) color	6 Increments, from green to yellow
LENT	Extent of lenticels	10 Increments from large to absent
PCOL	Extent of red color	10 Increments, from 5% to 95%
RUSS	Extent of russetting	11 Increments, from severe to absent
SHAPE	Shape	10 Increments, from flat to cylindrical
SIZE	Size	11 Increments, from tiny to very large
TCOL	Red color type	6 Increments, from blush to stripe
		
*Ordinal sensory traits*		
AROM	Aromatic flavor	12 Increments from none to very fruity
CRISP	Crispness	10 Increments from chewy to crisp
EQ	Eating quality summary	15 Increments from yuck to outstanding
HARD	Hardness	11 Increments from soft to very firm
JUIC	Juiciness	11 Increments from very dry to very juicy
OVERALL	Overall	4 Increments from reject to advance
SWEET	Sweetness	9 Increments
TART	Tartness	11 Increments

**Table 6 tbl6:** Genetic correlation matrix for apple fruit quality traits assessed following short-term storage among (a) instrumental traits M1, M2 and CN, and sensory traits HARD, CRISP and JUIC, and (b) instrumental traits SSC and TAI, and sensory traits SWEET, AROM and TART.

*(a)*	*HARD*	*CN*	*CRISP*	*JUIC*
M1	0.96	0.31	0.17	0.01
HARD		0.41	0.30	0.14
CN			0.75	0.71
CRISP				0.87

*(b)*	*AROM*	*SWEET*	*TA*	*TART*
SSC	0.56	0.57	0.12	0.25
AROM		0.84	0.05	0.25
SWEET			−0.29	−0.06
TA				0.97

## Appendix 1

[Table tbl5]

## Appendix 2

Derivation of expression for predicting response to selection

Falconer presents response to selection as the multiplication of selection intensity (*i*), narrow-sense heritability (*h*^*2*^) and s.e. of the phenotype (*σ_P_*).

$$R=ih^{2}\sigma_{P}$$

Heritability may be defined as the correlation between the selection clue and the true genetic value of the selection objective. For example, individual narrow sense heritability (*h*^2^) may be written as $\frac{\sigma_{A}^{2}}{\sigma_{P}^{2}}$, which is the correlation between the phenotype of an individual and the true additive genetic value of the individual. $\frac{\sigma_{A}^{2}}{\sigma_{P}^{2}}$ can be expanded to $\frac{\sigma_{A}*\sigma_{A}}{\sigma_{P}*\sigma_{P}}$, so that response to selection becomes: $R=i\frac{\sigma_{A}*\sigma_{A}}{\sigma_{P}*\sigma_{P}}\sigma_{P}$

The numerator *σ_P_* would cancel out a denominator *σ_P_*. Hence, response to selection on the phenotype to improve the additive genetic value can be written as: $R=i\frac{\sigma_{A}^{2}}{\sigma_{P}}$

When referring the mean of a genotype rather than of an individual, $\sigma_{\hat{G}}$ is used for phenotypic partitioning of the mean of a genotype, rather than *σ_P_*. $\sigma_{\hat{G}}$ is the predicted candidate effect or the selection clue. Additive variance can be rewritten as covariance between the predicted candidate effect and the true clonal mean of the candidate, or selection objective. Therefore, response to selection was calculated as: $R=i\frac{\tau_{\hat{G},G}}{\sigma_{\hat{G}}}$

## Appendix 3

Derivation of expression for predicting correlated response to selection

Falconer presents the equation for correlation response between two traits as:

$$CRS_{y}=ih_{x}r_{xy}\sigma_{Gy}$$

The heritability of trait *x* is $\frac{\sigma_{Gx}}{\sigma_{\hat{G}x}}$ and the genetic correlation of trait *x* and *y* is $r_{xy}=\frac{cov_{xy}}{\sqrt{\sigma_{Gx}^{2}\sigma_{Gy}^{2}}}$.

The square root of a variance turns into the s.e. of the estimate. The equation then becomes:

$$CRS_{y}=i\frac{\sigma_{Gx}}{\sigma_{\hat{G}x}}\frac{cov_{xy}}{\sigma_{Gx}\sigma_{Gy}}\sigma_{Gy}$$

The denominator of the genetic correlation is canceled with the same terms in the numerator, so the equation becomes:

$$CRS_{y}=i\frac{cov_{GxGy}}{\sigma_{\hat{G}x}}$$

Covariance of *Gx* and *Gy* can be rewritten as $rG_{x}G_{y}\sqrt{{\sigma^{2}}_{G_{x}}{\sigma^{2}}_{{\hat{G}}_{y}}}$

The equation is thus:

$$CRS_{y}=i\frac{rG_{x}G_{y}\sqrt{{\sigma^{2}}_{G_{x}}{\sigma^{2}}_{{\hat{G}}_{y}}}}{\sigma_{{\hat{G}}_{x}}}$$

## Appendix 4

[Table tbl6]
